# The almost sure local central limit theorem for products of partial sums under negative association

**DOI:** 10.1186/s13660-018-1875-8

**Published:** 2018-10-10

**Authors:** Yuanying Jiang, Qunying Wu

**Affiliations:** College of Science, Guilin University of Technology, Guilin, China

**Keywords:** 60E15, 60F15, Negative association, Products of partial sums, Almost sure local central limit theorem, Almost sure global central limit theorem

## Abstract

Let $\{X_{n}, n\geq1\}$ be a strictly stationary negatively associated sequence of positive random variables with $\mathrm{E}X_{1}=\mu>0$ and $\operatorname{Var}(X_{1})=\sigma^{2}<\infty$. Denote $S_{n}=\sum_{i=1}^{n}X_{i}, p_{k}=\mathrm{P}(a_{k}\leq ({\prod}_{j=1}^{k}S_{j}/(k!\mu^{k}) )^{1/(\gamma\sigma_{1} \sqrt{k})}< b_{k})$ and $\gamma=\sigma/\mu$ the coefficient of variation. Under some suitable conditions, we derive the almost sure local central limit theorem

$$\lim_{n\rightarrow\infty}\frac{1}{\log n}\sum_{k=1}^{n} \frac{1}{kp_{k}}\mathrm{I} \biggl\{ a_{k}\leq \biggl(\frac {\prod_{j=1}^{k}S_{j}}{k!\mu^{k}} \biggr)^{1/(\gamma\sigma_{1} \sqrt {k})}< b_{k} \biggr\} =1 \quad\mbox{a.s.,} $$

where $\sigma_{1}^{2}=1+\frac{1}{\sigma^{2}}\sum_{j=2}^{\infty}\operatorname{Cov}(X_{1},X_{j})>0$.

## Introduction

### Definition 1.1

([1])

A finite family of random variables $X_{1},X_{2},\ldots,X_{n},n\geq2$, is said to be negatively associated (NA) if, for every pair of disjoint subsets *A* and *B* of $\{1,2,\ldots,n\}$, we have

$$\operatorname{Cov}\bigl(f_{1}(X_{i}, i\in A),f_{2}(X_{j}, j\in B)\bigr)\leq0, $$

where $f_{1}$ and $f_{2}$ are coordinatewise increasing and the covariance exists. An infinite family of random variables (r.v.) is NA if every finite subfamily is NA.

Obviously, if $\{X_{i}, i\geq1\}$ is NA, and $\{f_{i}, i\geq1\}$ is a sequence of nondecreasing (or nonincreasing) functions, then $\{f_{i}(X_{i}), i\geq1\}$ is also NA. We refer to Roussas [2] for NA’s fundamental properties and applications in several fields, Shao [3] for the moment inequalities, Jing and Liang [4] and Cai [5] for the strong limit theorems, Chen et al. [6] and Sung [7] for the complete convergence.

Let $S_{n}:=\sum_{i=1}^{n}X_{i}$ denote the partial sum of $\{X_{i}, i\geq1\} $ and $\prod_{j=1}^{n}S_{j}$ is known as a product of partial sum $S_{j}$, the study on partial sum has received extensive attention. Such well-known classic laws as the central limit theorem (CLT), the almost sure central limit theorem (ASCLT), and law of the iterated logarithm (LIL) are known for characterizing the asymptotic behavior of $S_{n}$. However, the study of asymptotic behavior for product of partial sum is not so far, it was initiated by Arnold and Villaseñor [8]. This paper intends to study the limit behavior of product $\prod_{j=1}^{n}S_{j}$ under negative association.

Let $\{X_{n}, n\geq1\}$ be a strictly stationary NA sequence of positive r.v. with $\mathrm{E}X_{1}=\mu>0$, $\operatorname{Var}(X_{1})=\sigma^{2}<\infty$, and the coefficient of variance $\gamma=\sigma/\mu$. Assume that

1.1 $$\begin{aligned} & \bigl\vert \operatorname{Cov}(X_{1},X_{n+1}) \bigr\vert =O\bigl(n^{-1}(\log n)^{-2-\varepsilon}\bigr),\quad \mbox{for some } \varepsilon>0, \end{aligned}$$

1.2 $$\begin{aligned} &\sigma_{1}^{2}=1+\frac{1}{\sigma^{2}}{\sum }_{j=2}^{\infty}\operatorname {Cov}(X_{1},X_{j})>0. \end{aligned}$$

1. Li and Wang [9] obtained the following version of the CLT:
  1.3 $$ \biggl(\frac{\prod_{j=1}^{n}S_{j}}{n!\mu^{n}} \biggr)^{1/(\gamma\sigma_{1} \sqrt{n})} \stackrel{\mathrm{d}}{ \rightarrow} \exp(\sqrt{2}\mathcal {N}), \quad \mbox{as } n\rightarrow \infty, $$
  where $\mathcal{N}$ is a standard normal distribution random variable.
2. Li and Wang [10] proved the following ASCLT:
  1.4 $$ \lim_{n\rightarrow\infty}\frac{1}{\log n}\sum_{k=1}^{n} \frac{1}{k}\mathrm{I} \biggl\{ \biggl(\frac{\prod_{j=1}^{k}S_{j}}{k!\mu^{k}} \biggr)^{1/(\gamma\sigma_{1} \sqrt{k})}\leq x \biggr\} =F(x)\quad \mbox{a.s. for all }x\in \mathbb{R}, $$

here and elsewhere, $\mathrm{I}\{A\}$ represents the indicative function of the event *A* and $F(\cdot)$ is the distribution function of the log-normal random variable $\exp(\sqrt {2}\mathcal{N})$.

The almost sure central limit theorem was proposed by Brosamler [11] and Schatte [12]. In recent years, the ASCLT has been extensively studied, and an attractive research direction is to prove it under associated or dependent situations. There are some literature works for $\alpha, \rho, \phi$-mixing and associated random variables, we refer to Matuła [13], Lin [14], Zhang et al. [15], Matuła and Stȩpień [16], Hwang [17], Li [18], Miao and Xu [19], Wu and Jiang [20].

A more general version of ASCLT for products of partial sums was proved by Weng et al. [21]. The following theorem is due to them.

### Theorem A

*Let* $\{X_{n},n\geq1\}$ *be a sequence of independent and identically distributed positive random variables with* $\mathrm{E}X_{1}^{3}<\infty, \mathrm{E}X_{1}=\mu,\operatorname{Var}(X_{1})=\sigma^{2}, \gamma=\sigma/\mu$. $a_{k}, b_{k}$ *satisfy*

1.5 $$ 0 \leq a_{k} \leq1 \leq b_{k}\leq\infty,\quad k=1,2,\ldots $$

*Let*

1.6 $$ p_{k}:=\mathrm{P} \Bigl(a_{k}\leq \Bigl({\prod }_{j=1}^{k}S_{j}/\bigl(k!\mu^{k} \bigr) \Bigr)^{1/(\gamma\sqrt{k})}< b_{k} \Bigr) $$

*and assume for sufficiently large k*, $p_{k}\geq1/(\log k)^{\delta_{1}}$ *for some* $\delta_{1}>0$. *Then we have*

1.7 $$ \lim_{n\rightarrow\infty}\frac{1}{\log n}\sum_{k=1}^{n} \frac{1}{kp_{k}}\mathrm{I} \biggl\{ a_{k}\leq \biggl(\frac {\prod_{j=1}^{k}S_{j}}{k!\mu^{k}} \biggr)^{1/(\gamma\sqrt{k})}< b_{k} \biggr\} =1 \quad \textit{a.s.} $$

This result may be called almost sure local central limit theorem (ASLCLT) for the product $\prod_{j=1}^{n}S_{j}$ of independent and identically distributed positive r.v., while (1.4) may be called almost sure global central limit theorem (ASGCLT).

The ASLCLT for partial sums of independent and identically distributed r.v. was stimulated by Csáki et al. [22], and Khurelbaatar [23] extended it to the case of *ρ*-mixing sequences, Jiang and Wu [24] extended it to the case of NA sequences. Zang [25] obtained the ASLCLT for a sample range.

In this paper, our concern is to give a common generalization of (1.7) to the case of NA sequences. The remainder of the paper is organized as follows. Section 2 provides our main result. Section 3 gives some auxiliary lemmas. The proofs of the theorem and some lemmas are in Sect. 4.

## Main results

In the following, we assume that $\{X_{n},n\geq1\}$ is a strictly stationary negatively associated sequence of positive r.v.’s with $\mathrm{E}X_{1}=\mu>0,\operatorname{Var}(X_{1})=\sigma^{2}<\infty,\mathrm {E}X_{1}^{3}<\infty$, the coefficient of variation $\gamma=\sigma/\mu$. $a_{k}, b_{k}$ satisfy

2.1 $$ 0 \leq a_{k} \leq1 \leq b_{k}\leq\infty,\quad k=1,2,\ldots $$

and

2.2 $$\begin{aligned} &\sigma_{1}^{2}:=1+\frac{1}{\sigma^{2}}{\sum }_{j=2}^{\infty}\operatorname {Cov}(X_{1},X_{j}) , \end{aligned}$$

2.3 $$\begin{aligned} &p_{k}:=\mathrm{P} \Bigl(a_{k}\leq \Bigl({\prod }_{j=1}^{k}S_{j}/\bigl(k!\mu^{k} \bigr) \Bigr)^{1/(\gamma\sigma_{1}\sqrt{k})}< b_{k} \Bigr). \end{aligned}$$

Then we study the asymptotic behavior of the logarithmic average

2.4 $$ \frac{1}{\log n}\sum_{k=1}^{n} \frac{1}{kp_{k}}\mathrm{I} \biggl\{ a_{k}\leq \biggl(\frac {\prod_{j=1}^{k}S_{j}}{k!\mu^{k}} \biggr)^{1/(\gamma\sigma_{1} \sqrt {k})}< b_{k} \biggr\} , $$

where the expression in the sum above is defined to be one if the denominator is zero. That is, let $\{a_{n},n\geq1\}$ and $\{b_{n},n\geq1\}$ be two sequences of real numbers and

2.5 $$ \alpha_{k}:= \textstyle\begin{cases} \frac{1}{p_{k}}\mathrm{I} \{a_{k}\leq (\frac{\prod_{j=1}^{k}S_{j}}{k!\mu^{k}} )^{1/(\gamma\sigma_{1} \sqrt{k})}< b_{k} \} ,&\mbox{if } p_{k}\neq0,\\ 1 ,& \mbox{if } p_{k}=0. \end{cases} $$

Therefore, we should study the asymptotic limit properties of $\frac {1}{\log n}\sum_{k=1}^{n}\frac{\alpha_{k}}{k}$ under suitable conditions.

In the following discussion, we shall use the definition of the Cox–Grimmett coefficient

2.6 $$ u(n):=\sup_{k\in\mathbb{N}}\sum_{j: \vert j-k \vert \geq n} \bigl\vert \operatorname{Cov}(X_{j},X_{k}) \bigr\vert , \quad n\in\mathbb{N}\cup\{0\}, $$

and we can verify that the formula

2.7 $$ u(n)=-2\sum_{k=n+1}^{\infty}\operatorname{Cov}(X_{1},X_{k}), \quad n\in\mathbb{N} $$

is correct for a stationary sequence of negatively associated random variables.

In the following, $\xi_{n}\sim\eta_{n}$ denotes $\xi_{n}/\eta_{n}\rightarrow1$, $n\rightarrow\infty$. $\xi_{n}=O(\eta_{n})$ denotes that there exists a constant $c>0$ such that $\xi_{n}\leq c\eta_{n}$ for sufficiently large *n*. The symbols $c, c_{1}, c_{2}, \ldots $ represent generic positive constants.

### Theorem 2.1

*Let* $\{X_{n},n\geq1\}$ *be a strictly stationary negatively associated sequence of positive r*.*v*. *with* $\mathrm {E}X_{1}=\mu>0,\operatorname{Var}(X_{1})=\sigma^{2}<\infty,\mathrm {E}X_{1}^{3}<\infty, \gamma=\sigma/\mu$. $a_{k}, b_{k}$ *satisfy* (2.1), *assume that* (1.1) *and* (1.2) *hold*, *and*

2.8 $$ \sum_{n=1}^{\infty} u(n)< \infty, $$

*and*

2.9 $$ p_{k}\geq\frac{1}{(\log k)^{\delta_{1}}} $$

*for sufficiently large* *k* *and some* $0<\delta_{1}<1/4$. *Then we have*

2.10 $$ \lim_{n\rightarrow\infty}\frac{1}{\log n}\sum_{k=1}^{n} \frac{\alpha _{k}}{k} =1,\quad \textit{a.s.,} $$

*where* $\alpha_{k}$ *is defined by* (2.5).

### Remark 2.2

Let $a_{k}=0$ and $b_{k}=x$ in (2.3). By CLT (1.3), we have

$$p_{k}=\mathrm{P} \Bigl( \Bigl({\prod}_{j=1}^{k}S_{j}/ \bigl(k!\mu^{k}\bigr) \Bigr)^{1/(\gamma \sigma_{1}\sqrt{k})}\leq x \Bigr) \rightarrow \mathrm{P} \bigl(\exp(\sqrt {2}\mathcal{N})\leq x \bigr)=F(x), \quad \mbox{as }k \rightarrow\infty. $$

Obviously (2.9) holds, then (2.10) becomes (1.4), which is the ASGCLT. Thus the ASLCLT is a general result which contains the ASGCLT.

## Auxiliary lemmas

In order to prove the main theorem, we need to use the concept of a triangular array of random variables. Let $b_{k,n}=\sum_{i=k}^{n}1/i$ and $Y_{i}=(X_{i}-\mu)/\sigma$. We define a triangular array $Z_{1,n},Z_{2,n},\ldots,Z_{n,n}$ as $Z_{k,n}=b_{k,n}Y_{k}$ and put $S_{k,n}=Z_{1,n}+Z_{2,n}+\cdots+Z_{k,n}$ for $1\leq k\leq n$. Let

3.1 $$\begin{aligned} U_{k} :=&\frac{1}{\gamma\sigma_{1}\sqrt{2k}}\sum_{i=1}^{k} \log\frac{S_{i}}{i\mu } \\ =&\frac{1}{\gamma\sigma_{1}\sqrt{2k}}\sum_{i=1}^{k} \biggl(\frac{S_{i}}{i\mu }-1 \biggr)+T_{k} \\ =& \frac{1}{\sigma_{1}\sqrt{2k}}S_{k,k}+T_{k}, \end{aligned}$$

where

3.2 $$ T_{k}=\frac{1}{\gamma\sigma_{1}\sqrt{2k}}\sum_{i=1}^{k} \frac{(S_{i}/i\mu -1)^{2}}{(1+\theta(S_{i}/i\mu-1))^{2}}, \quad \vert \theta \vert \leq1. $$

Note that, for $l>k$, we have

$$\begin{aligned} S_{l,l}-S_{k,k} =&\sum_{j=1}^{l}b_{j,l}Y_{j}- \sum_{j=1}^{k}b_{j,k}Y_{j}=b_{k+1,l}(Y_{1}+ \cdots+Y_{k})+(b_{k+1,l}Y_{k+1}+\cdots+b_{1,l}Y_{l}) \\ =&b_{k+1,l}\widetilde{S}_{k}+(b_{k+1,l}Y_{k+1}+ \cdots+b_{1,l}Y_{l}). \end{aligned}$$

So, by the property of NA sequences, $S_{l,l}-S_{k,k}-b_{k+1,l}\widetilde{S}_{k}$ and $U_{k}$ are negatively associated.

The following Lemma 3.1 is due to Liang et al. [26].

### Lemma 3.1

*Let* $\{X_{n},n\geq1\}$ *be a sequence of NA random variables with* $\mathrm{E}X_{n}=0$ *and* $\{a_{ni},1\leq i\leq n,n\geq1\}$ *be an array of real numbers such that* $\sup_{n}\sum_{i=1}^{n}a_{ni}^{2}<\infty$ *and* $\max_{1\leq i\leq n}|a_{ni}|\rightarrow0$ *as* $n\rightarrow\infty$. *Assume that* $\sum_{j:|k-j|\geq n}|\operatorname{Cov}(X_{k},X_{j})|\rightarrow0$ *as* $n\rightarrow\infty$ *uniformly for* $k\geq1$. *If* $\operatorname{Var}(\sum_{i=1}^{n}a_{ni}X_{i})=1$ *and* $\{X_{n}^{2},n\geq1 \}$ *is a uniformly integrable family*, *then* $\sum_{i=1}^{n}a_{ni}X_{i} \stackrel{\mathrm {d}}{\rightarrow} \mathcal{N}$, *where* $\mathcal{N}$ *is a standard normal distribution random variable*.

Now we obtain the CLT for triangular arrays.

### Lemma 3.2

*Let* $\{Y_{n},n\geq1\}$ *be a strictly stationary sequence of negatively associated random variables with* $\mathrm{E}Y_{1}=0, \operatorname{Var}(Y_{1})=1$ *and* $\sigma _{1}^{2}=1+\sum_{j=2}^{\infty}\operatorname{Cov}(Y_{1},Y_{j})>0$. *Suppose that there exist constants* $\delta_{2}$ *and* $\delta_{3}$ *such that* $0 < \delta _{2}, \delta_{3} < 1$. *Assume also that* (1.1) *and* (1.2) *hold*. *If*

3.3 $$ \log l>(\log n)^{\delta_{2}}, \quad k< \frac{l}{(\log l)^{2+\delta _{3}}} $$

*for sufficiently large* *n*, *then*

3.4 $$ \frac{1}{\sigma_{1}\sqrt{2l-2k}}\sum_{j=k+1}^{l}b_{j,l}Y_{j} \stackrel {\mathrm{d}}{\rightarrow} \mathcal{N } \quad \textit{as } n\rightarrow \infty. $$

The proof is quite long and will be left to Sect. 4.

The following Lemma 3.3 is a corollary to Corollary 2.2 in Matuła [27] under a strictly stationary condition.

### Lemma 3.3

*If the conditions of Lemma *3.2 *and* (2.8) *hold*, *assume also* $\mathrm{E}|Y_{1}|^{3}<\infty$. *Let*

$$ F_{n}(y):=\mathrm{P} \biggl(\frac{\sum_{j=1}^{n}b_{j,n}Y_{j} }{\sigma_{1}\sqrt{2n}}< y \biggr), \qquad F_{k,l}(y):=\mathrm{P} \biggl(\frac{\sum_{j=k+1}^{l}b_{j,l}Y_{j} }{\sigma_{1}\sqrt{2l-2k}}< y \biggr). $$

*Then we have*

3.5 $$ \sup_{y\in\mathbb{R}} \bigl\vert F_{n}(y)-\varPhi(y) \bigr\vert =O\bigl(n^{-1/5}\bigr) $$

*and*

3.6 $$ \sup_{y\in\mathbb{R}} \bigl\vert F_{k,l}(y)-\varPhi(y) \bigr\vert =O\bigl((l-k)^{-1/5}\bigr). $$

### Lemma 3.4

*If the conditions of Theorem* 2.1 *hold*, *and assume that there exists* $\delta_{4}$ *such that* $0<\delta_{1}<\delta_{4}<1/4$. *Let* $\varepsilon_{l}=1/(\log l)^{\delta_{4}}$, *where* $l=3,4,\ldots,n$, *then the following asymptotic relations hold*:

3.7 $$\begin{aligned} &\sum_{\substack{ \mathcal{H} }}\frac{1}{kl(l-k)^{1/5}p_{l}}=O\bigl((\log n)^{2-\epsilon}\bigr), \end{aligned}$$

3.8 $$\begin{aligned} &\sum_{\substack{ \mathcal{H} }}\frac{1}{l^{3/2}\sqrt{l-k}p_{l}}=O\bigl((\log n)^{2-\epsilon}\bigr), \end{aligned}$$

3.9 $$\begin{aligned} &\sum_{\substack{ \mathcal{H} }}\frac{\varepsilon_{l}}{k\sqrt{l}\sqrt{l-k}p_{l}}=O\bigl((\log n)^{2-\epsilon }\bigr), \end{aligned}$$

3.10 $$\begin{aligned} &\sum_{\substack{ \mathcal{H} }}\frac{1}{klp_{k}p_{l}}\mathrm{P} \biggl\{ \biggl\vert \frac{1}{\sigma_{1}\sqrt {2l}}S_{k,k} \biggr\vert \geq\varepsilon _{l} \biggr\} =O\bigl((\log n)^{2-\epsilon}\bigr), \end{aligned}$$

3.11 $$\begin{aligned} &\sum_{\substack{ \mathcal{H} }}\frac{1}{klp_{k}p_{l}}\mathrm{P} \biggl\{ \biggl\vert \frac{1}{\sigma_{1}\sqrt {2l}}b_{k+1,l}\widetilde{S}_{k} \biggr\vert \geq\varepsilon _{l} \biggr\} =O\bigl((\log n)^{2-\epsilon} \bigr), \end{aligned}$$

3.12 $$\begin{aligned} &\sum_{\substack{ \mathcal{H} }}\frac{1}{klp_{k}p_{l}}\mathrm{P} \bigl\{ \vert T_{l} \vert \geq\varepsilon _{l} \bigr\} =O\bigl(( \log n)^{2-\epsilon}\bigr), \end{aligned}$$

*where* $\mathcal{H}:=\{(k,l):1\leq k< l\leq n, \log l>(\log n)^{\delta_{2}}\textit{ and }k< l/(\log l)^{2+\delta_{3}}\}$ *and* $0<\epsilon<1-2(\delta_{1}+\delta_{4})$.

The proof will be left to Sect. 4.

The following result is due to Khurelbaatar [23].

### Lemma 3.5

*Assume that* $\{\xi_{n}, n\geq1\}$ *is a non*-*negative random sequence such that* $\mathrm{E}\xi_{k}=1, k=1,2,\ldots $ , *and*

3.13 $$ \operatorname{Var} \Biggl(\sum_{k=1}^{n} \frac{\xi_{k}}{k} \Biggr)\leq c (\log n)^{2-\epsilon}, $$

*for some* $\epsilon>0$ *and positive constant* *c*, *then*

3.14 $$ \lim_{n\rightarrow\infty}\frac{1}{\log n}\sum_{k=1}^{n} \frac{1}{k}\xi_{k}=1 \quad \textit{a.s.} $$

The following Lemma 3.6 is obvious.

### Lemma 3.6

*Assume that the non*-*negative random sequence* $\{\xi_{n}, n\geq1\}$ *satisfies* (3.14) *and the sequence* $\{\eta_{n}, n\geq1\}$ *is such that*, *for any* $\varepsilon>0$, *there exists* $k_{0}=k_{0}(\varepsilon,\omega)$ *for which*

$$ (1-\varepsilon)\xi_{k}\leq\eta_{k}\leq(1+\varepsilon) \xi_{k}, \quad k>k_{0}. $$

*Then we also have*

$$ \lim_{n\rightarrow\infty}\frac{1}{\log n}\sum_{k=1}^{n} \frac{1}{k}\eta_{k}=1 \quad \textit{a.s.} $$

## Proofs of the main result and lemmas

The main aspect of our proof of Theorem 2.1 is verification condition (3.13) for $\alpha_{k}$, where $\alpha_{k}$ is defined by (2.5). We use ASCLT (1.4) with remainders and the following elementary inequalities:

4.1 $$ \bigl\vert \varPhi(x)-\varPhi(y) \bigr\vert \leq c \vert x-y \vert \quad \mbox{for every } x,y\in\mathbb{R}, $$

with some constant *c*. Moreover, for each $k > 0$, there exists $c_{1}= c_{1}(k)$ such that

4.2 $$ \bigl\vert \varPhi(x)-\varPhi(y) \bigr\vert \geq c_{1} \vert x-y \vert \quad \mbox{for every } x,y\in\mathbb{R}\mbox{ and } \vert x \vert + \vert y \vert \leq k. $$

### Proof of Theorem 2.1

Let

4.3 $$ \hat{a}_{k}=\frac{1}{\sqrt{2}\log a_{k}},\qquad \hat{b}_{k}= \frac{1}{\sqrt{2}\log b_{k}},\quad k=1,2,\ldots $$

Thus, $-\infty\leq\hat{a}_{k}\leq0\leq\hat{b}_{k}\leq\infty$ by (2.1). By the definition of $U_{k}$ in (3.1), we have $p_{k}=\mathrm{P}(\hat{a}_{k}\leq U_{k}<\hat{b}_{k})$ and

4.4 $$ \alpha_{k}:= \textstyle\begin{cases} \frac{\mathrm{I}\{\hat{a}_{k}\leq U_{k}< \hat{b}_{k}\}}{p_{k}} ,&\mbox{if } p_{k}\neq0,\\ 1 ,& \mbox{if } p_{k}=0. \end{cases} $$

First assume that

4.5 $$ b_{k}-a_{k}\leq c ,\quad k=1,2,\ldots, $$

with some constant *c*. Note that

4.6 $$\begin{aligned} \operatorname{Var} \Biggl(\sum _{k=1}^{n}\frac{\alpha_{k}}{k} \Biggr) =&\sum _{k=1}^{n}\frac{\operatorname{Var}(\alpha_{k})}{k^{2}} +2\sum _{\substack{ 1\leq k< l\leq n}}\frac{ \operatorname{Cov}(\alpha_{k},\alpha_{l})}{kl} \\ =&\sum_{k=1}^{n} \frac{\operatorname{Var}(\alpha_{k})}{k^{2}} +2 \biggl[\sum_{\substack{ 1\leq k< l\leq n\\ \log l\leq(\log n)^{\delta_{2}} }} +\sum _{\substack{ 1\leq k< l\leq n\\ \log l>(\log n)^{\delta_{2}}\\ k>l/(\log l)^{2+\delta_{3}} }} +\sum_{\substack{ 1\leq k< l\leq n\\ \log l>(\log n)^{\delta_{2}}\\ k\leq l/(\log l)^{2+\delta_{3}} }} \biggr] \frac{\operatorname{Cov}(\alpha_{k},\alpha_{l})}{kl} \\ :=&{\sum}_{1}+{\sum}_{2}+{\sum }_{3}+{\sum}_{4}, \end{aligned}$$

where $\delta_{2},\delta_{3}$ are defined by Lemma 3.2. Note also that $\operatorname{Var}(\alpha_{k})=0$ if $p_{k}= 0$ and

$$ \operatorname{Var}(\alpha_{k})=\frac{1-p_{k}}{p_{k}}\leq \frac {1}{p_{k}} \quad \mbox{if } p_{k}\neq0. $$

And by the condition of (2.9), we have

4.7 $$\begin{aligned} \sum\nolimits_{1} \leq&\sum_{\substack{ 1\leq k\leq n\\ p_{k} \neq0 }} \frac{1}{k^{2}p_{k}}\leq c(\log n)^{2-\epsilon}. \end{aligned}$$

If either $p_{k}=0$ or $p_{l}=0$, then obviously $\operatorname{Cov}(\alpha _{k},\alpha_{l})=0$, so we may assume that $p_{k}p_{l}\neq0$, by (2.1), we have

4.8 $$\begin{aligned} {\sum}_{2} =&2\sum_{\substack{ 1\leq k< l\leq n\\ \log l\leq(\log n)^{\delta_{2}} }} \frac{1}{kl} \frac{\mathrm{P}(\hat{a}_{k}\leq U_{k}< \hat{b}_{k},\hat {a}_{l}\leq U_{l}< \hat{b}_{l})- p_{k}p_{l}}{p_{k}p_{l}} \\ \leq&2\sum_{\substack{ 1\leq k< l\leq n\\ \log l\leq(\log n)^{\delta_{2}} }}\frac{1}{kl} \frac{1- p_{k}}{p_{k}}\leq2\sum_{\substack{ 1\leq k< l\leq n\\ \log l\leq(\log n)^{\delta_{2}} }}\frac{1}{kl} \frac{1}{p_{k}} \\ \leq&2(\log n)^{\delta_{1}+2\delta_{2}}\leq c(\log n)^{2-\epsilon} \end{aligned}$$

for $\delta_{1}<1/4$ and $\delta_{2}<7/8$. Now we estimate the bound of ∑_3_. Let $A_{n}$ be an integer such that $\log A_{n} \sim(\log n)^{\delta_{2}}$ for sufficiently large *n*. Then

4.9 $$\begin{aligned} {\sum}_{3} \leq&2\sum_{l=A_{n}}^{n} \sum_{k=l/(\log l)^{2+\delta _{3}}}^{l-1}\frac{1}{kl} \frac{1}{p_{k}} \\ \leq&2(\log n)^{\delta_{1}} \Biggl[\sum _{l=A}^{n}\frac{1}{l}\frac {(\log l)^{2+\delta_{3}}}{l}+\sum _{l=A}^{n}\frac{1}{l}\sum _{k=1+l/(\log l)^{2+\delta_{3}}}^{l}\frac{1}{k} \Biggr] \\ \leq&c(\log n)^{\delta_{1}}\sum_{l=A}^{n} \frac{1}{l}\log(\log l)^{2+\delta_{3}} \\ \leq& c(\log n)^{2-\epsilon}. \end{aligned}$$

So, it remains to estimate the bound of ∑_4_. Let $1\leq k< l$ and $\varepsilon_{l}=1/(\log l)^{\delta_{4}}$, where $0<\delta_{1}<\delta _{4}<1/4$, we have

$$\begin{aligned} &\operatorname{Cov}(\alpha_{k},\alpha_{l}) \\ &\quad = \frac{1}{p_{k} p_{l}}\operatorname{Cov} \bigl(I\{\hat{a}_{k}\leq U_{k}< \hat{b}_{k}\},I\{\hat{a}_{l}\leq U_{l}< \hat {b}_{l}\} \bigr) \\ &\quad =\frac{1}{p_{k} p_{l}} \biggl[\mathrm{P} \biggl\{ \hat{a}_{k}\leq U_{k}< \hat{b}_{k},\hat{a}_{l}\leq \frac{1}{\sigma_{1}\sqrt{2l}}S_{l,l}+T_{l}< \hat {b}_{l} \biggr\} \\ &\qquad {}-\mathrm{P}\{\hat{a}_{k}\leq U_{k}< \hat{b}_{k}\} \mathrm{P} \biggl\{ \hat{a}_{l}\leq \frac{1}{\sigma_{1}\sqrt {2l}}S_{l,l}+T_{l}< \hat{b}_{l} \biggr\} \biggr] \\ &\quad \leq \frac{1}{p_{k} p_{l}} [\mathrm{P} \biggl\{ \hat{a}_{k}\leq U_{k}< \hat{b}_{k},\hat{a}_{l}-3 \varepsilon_{l}\leq\frac{1}{\sigma_{1}\sqrt {2l}}(S_{l,l}-S_{k,k}-b_{k+1,l} \tilde{S}_{k})< \hat{b}_{l}+3\varepsilon_{l} \biggr\} \\ &\qquad {}+2\mathrm{P} \biggl\{ \biggl\vert \frac{1}{\sqrt {2l}}S_{k,k} \biggr\vert \geq\varepsilon_{l} \biggr\} +2\mathrm{P} \biggl\{ \biggl\vert \frac {1}{\sigma_{1}\sqrt{2l}}b_{k+1,l}\tilde{S}_{k} \biggr\vert \geq\varepsilon_{l} \biggr\} +2\mathrm{P} \bigl\{ \vert T_{l} \vert \geq\varepsilon_{l} \bigr\} \\ &\qquad {}-\mathrm{P}\{\hat{a}_{k}\leq U_{k}< \hat{b}_{k}\} \biggl[\mathrm{P} \biggl\{ \hat{a}_{l}- \varepsilon_{l}\leq\frac{1}{\sigma_{1}\sqrt {2l}}S_{l,l}< \hat{b}_{l}+\varepsilon_{l} \biggr\} -2\mathrm{P} \bigl\{ \vert T_{l} \vert \geq\varepsilon_{l} \bigr\} \biggr] \} \\ &\quad \leq \frac{1}{p_{l}}B_{1}+B_{2}, \end{aligned}$$

where

$$\begin{aligned} B_{1}={}&\mathrm{P} \biggl\{ \hat{a}_{l}-3 \varepsilon_{l}\leq\sqrt{1-\frac{k}{l}} \frac{S_{l,l}-S_{k,k}-b_{k+1,l}\tilde{S_{k}}}{\sigma_{1}\sqrt{2l-2k}}< \hat {b}_{l}+3\varepsilon_{l} \biggr\} \\ &{}-\mathrm{P} \biggl\{ \hat{a}_{l}-\varepsilon _{l}\leq \frac{1}{\sigma_{1}\sqrt{2l}}S_{l,l}< \hat{b}_{l}+\varepsilon_{l} \biggr\} \end{aligned}$$

and

$$ B_{2}=\frac{1}{p_{k} p_{l}} \biggl[ 2\mathrm{P} \biggl\{ \biggl\vert \frac {S_{k,k}}{\sigma_{1}\sqrt{2l}} \biggr\vert \geq\varepsilon_{l} \biggr\} +2\mathrm {P} \biggl\{ \biggl\vert \frac{b_{k+1,l}\tilde{S}_{k}}{\sigma_{1}\sqrt{2l}} \biggr\vert \geq \varepsilon_{l} \biggr\} +4\mathrm{P} \bigl\{ \vert T_{l} \vert \geq \varepsilon_{l} \bigr\} \biggr]. $$

So by (3.3), Lemma 3.3, and (4.1), we obtain

$$\begin{aligned} B_{1} \leq& \biggl[F_{k,l} \biggl(\frac{\hat{b}_{l}+3\varepsilon _{l}}{\sqrt{1-k/l}} \biggr)-\varPhi \biggl(\frac{\hat{b}_{l}+3\varepsilon _{l}}{\sqrt{1-k/l}} \biggr) \biggr]- \biggl[F_{k,l} \biggl(\frac{\hat {a}_{l}-3\varepsilon_{l}}{\sqrt{1-k/l}} \biggr)-\varPhi \biggl(\frac{\hat {a}_{l}-3\varepsilon_{l}}{\sqrt{1-k/l}} \biggr) \biggr] \\ &{}+ \biggl[\varPhi \biggl(\frac{\hat{b}_{l}+3\varepsilon _{l}}{\sqrt{1-k/l}} \biggr)-\varPhi \biggl( \frac{\hat{a}_{l}-3\varepsilon _{l}}{\sqrt{1-k/l}} \biggr) \biggr]-\bigl[F_{l}(\hat{b}- \varepsilon_{l})-\varPhi(\hat {b}-\varepsilon_{l})\bigr] \\ &{}+\bigl[F_{l}(\hat{b}+\varepsilon_{l})-\varPhi(\hat {a}+ \varepsilon_{l})\bigr]-\bigl[\varPhi(\hat{b}-\varepsilon_{l})- \varPhi(\hat {a}-\varepsilon_{l})\bigr] \\ \leq&c\frac{1}{(l-k)^{1/5}}+\varPhi \biggl(\frac{\hat {b}_{l}+3\varepsilon_{l}}{\sqrt{1-k/l}} \biggr)-\varPhi \biggl(\frac{\hat {a}_{l}-3\varepsilon_{l}}{\sqrt{1-k/l}} \biggr)\\ &{}+\frac{c}{l^{1/5}}-\varPhi (\hat{b}- \varepsilon_{l})+\varPhi(\hat{a}+\varepsilon_{l}) \\ \leq&c\frac{1}{(l-k)^{1/5}}+ \biggl(\frac{\sqrt{l}}{\sqrt {l-k}}-1 \biggr) ( \hat{b}_{l}-\hat{a}_{l})+6\varepsilon_{l} \frac{\sqrt{l}}{\sqrt {l-k}}+2\varepsilon_{l} \\ \leq&c \biggl(\frac{1}{(l-k)^{1/5}}+\frac{k}{\sqrt {l(l-k)}}+\varepsilon_{l} \frac{\sqrt{l}}{\sqrt{l-k}} \biggr). \end{aligned}$$

So, by using Lemma 3.4, we have

4.10 $$ {\sum}_{4}\leq2\sum_{\substack{ 1\leq k< l\leq n\\ \log l>(\log n)^{\delta_{2}}\\ k\leq l/(\log l)^{2+\delta_{3}} }} \frac{1}{kl} \biggl(\frac{1}{p_{l}}B_{1}+B_{2} \biggr)\leq c(\log n)^{2-\epsilon}. $$

Combining (4.7)–(4.10) implies that

$$ \operatorname{Var} \Biggl(\sum_{k=1}^{n} \frac{\alpha_{k}}{k} \Biggr)\leq c(\log n)^{2-\epsilon}, \quad \mbox{as } n \rightarrow\infty. $$

Hence applying Lemma 3.5, our theorem is proved under the restricting condition (4.5).

Then, we remove the restricting condition (4.5). Fix $x>0$ and define

$$\begin{aligned} &\widetilde{a}_{k}=\max(a_{k}, - x),\\ &\widetilde{b}_{k}=\min(b_{k}, x), \\ &\widetilde{p}_{k}= \mathrm{P}(\widetilde{a}_{k}\leq U_{k}< \widetilde{b}_{k}). \end{aligned}$$

Clearly $\widetilde{b}_{k}-\widetilde{a}_{k}\leq\min(2x,c)$ and $\widetilde {p}_{k}\leq p_{k}$, so assuming $\widetilde{p}_{k}\neq0$, then we also have $p_{k}\neq0$, thus

4.11 $$\begin{aligned} \alpha_{k} =& \frac{1}{p_{k}}I \biggl\{ a_{k} \leq \biggl(\frac{\prod_{j=1}^{k}S_{j}}{k!\mu^{k}} \biggr)^{1/(\gamma\sigma_{1} \sqrt{k})}< b_{k} \biggr\} \\ =&\frac{1}{p_{k}} \bigl[I\{\widetilde{a}_{k}\leq U_{k}< \widetilde{b}_{k}\}+I\{a_{k}\leq U_{k}< \widetilde{a}_{k}\}+I\{\widetilde{b}_{k}\leq U_{k}< b_{k}\} \bigr] \\ \leq&\frac{1}{\widetilde{p}_{k}}I\{\widetilde{a}_{k}\leq U_{k}< \widetilde{b}_{k}\}+\frac{1}{{p_{k}}} \bigl[I \{a_{k}\leq U_{k}< \widetilde{a}_{k}\}+I\{ \widetilde{b}_{k}\leq U_{k}< b_{k}\} \bigr] \\ \leq&\frac{1}{\widetilde{p}_{k}}I\{\widetilde{a}_{k}\leq U_{k}< \widetilde{b}_{k}\}+\frac{I\{U_{k}< - x\}}{\mathrm{P}(- x\leq U_{k}< 0)}+\frac{I\{U_{k}\geq x\}}{\mathrm{P}(0\leq U_{k}< x)}. \end{aligned}$$

By the law of large numbers, we get $(\frac{S_{i}}{i\mu}-1)\stackrel {\mathrm{P}}{\rightarrow}0$. Noting that $x^{2}/(1+\theta x)^{2}\leq4x^{2}$ for $|x|<1/2$ and $\theta\in(0,1)$, and by using Markov’s inequality, $\forall\varepsilon>0$, we have

4.12 $$\begin{aligned} \mathrm{P}\bigl\{ \vert T_{k} \vert \geq\varepsilon\bigr\} =&\mathrm{P} \Biggl\{ \Biggl\vert \frac{1}{\gamma\sigma_{1}\sqrt{2k}}\sum _{i=1}^{k}\frac{(\frac{S_{i}}{i\mu }-1)^{2}}{(1+\theta(\frac{S_{i}}{i\mu}-1))^{2}} \Biggr\vert \geq \varepsilon \Biggr\} \\ \leq&\mathrm{P} \Biggl\{ \Biggl\vert \frac{4}{\gamma\sigma_{1}\sqrt{2k}}\sum _{i=1}^{k} \biggl(\frac{S_{i}}{i\mu}-1 \biggr)^{2} \Biggr\vert \geq\varepsilon \Biggr\} \\ \leq&\frac{2\sqrt{2}\sum_{i=1}^{k}\mathrm{E} (\frac {S_{i}}{i\mu}-1 )^{2}}{\gamma\sigma_{1}\sqrt{k}\varepsilon}\leq\frac {2\sqrt{2}\sum_{i=1}^{k}\frac{\sigma^{2}}{i^{2}\mu^{2}}\operatorname{Var} (\sum_{j=1}^{i}Y_{j} )}{\gamma\sigma_{1}\sqrt{k}\varepsilon} \\ \leq&\frac{2\sqrt{2}\sigma^{2}\sum_{i=1}^{k}\frac{1}{i}}{\gamma\mu ^{2}\sigma_{1}\sqrt{k}\varepsilon}\leq\frac{2\sqrt{2}\gamma\log k}{\sigma _{1}\sqrt{k}\varepsilon}. \end{aligned}$$

Then we have $T_{k}\stackrel{\mathrm{P}}{\rightarrow} 0 $ by (4.12) and $S_{k,k}/(\sigma_{1}\sqrt{2k})\stackrel{\mathrm {d}}{\rightarrow} \mathcal{N}$ by Lemma 2.4 of Li and Wang [10]. So, by Slutsky’s theorem, we have

4.13 $$ U_{k}=T_{k}+ \frac{1}{ \sigma_{1}\sqrt{2k}}S_{k,k} \stackrel{ \mathrm {d}}{\rightarrow} \mathcal{N }. $$

Thus, we obtain

4.14 $$ \lim_{k\rightarrow\infty}\mathrm{P}(- x\leq U_{k}< 0)=\varPhi(0)- \varPhi(-x) $$

and

4.15 $$ \lim_{k\rightarrow\infty}\mathrm{P}(0\leq U_{k}< x)=\varPhi(x)- \varPhi (0). $$

Applying ASCLT (1.4), i.e.,

4.16 $$ \lim_{n\rightarrow\infty}\frac{1}{\log n}\sum_{k=1}^{n} \frac{1}{k}\mathrm{I} \{U_{k}\leq x \}=\varPhi (x)\quad \mbox{a.s. for all }x\in\mathbb{R}, $$

and Lemma 3.6, (4.14), and (4.15), we obtain

4.17 $$ \lim_{n\rightarrow\infty} \frac{1}{\log n} \sum _{k=1}^{n} \frac{\mathrm {I}\{U_{k}< - x\}}{k\mathrm{P}(- x\leq U_{k}< 0)}=\frac{\varPhi(-x)}{\varPhi(0)-\varPhi(-x)}\quad \mbox{a.s.} $$

and

4.18 $$ \lim_{n\rightarrow\infty} \frac{1}{\log n} \sum _{k=1}^{n} \frac{\mathrm {I}\{U_{k}> x\}}{k\mathrm{P}(0\leq U_{k}< x)}=\frac{1-\varPhi(x)}{\varPhi(x)-\varPhi (0)}\quad \mbox{a.s.} $$

Since $\widetilde{a}_{k}$ and $\widetilde{b}_{k}$ satisfy (4.5), we get

4.19 $$ \lim_{n\rightarrow\infty}\frac{1}{\log n}\sum_{k=1}^{n} \frac{\widetilde{\alpha}_{k}}{k}=1 \quad \mbox{a.s.}, $$

where

$$ \widetilde{\alpha}_{k}= \textstyle\begin{cases} \frac{1}{\widetilde{p}_{k}}\mathrm{I}\{\widetilde{a}_{k}\leq U_{k}< \widetilde {b}_{k}\} ,& \mbox{if } \widetilde{p}_{k}\neq 0,\\ 1 ,& \mbox{if } \widetilde{p}_{k}=0. \end{cases} $$

Equations (4.11) and (4.17)–(4.19) together imply that

$$ \limsup_{n\rightarrow\infty}\frac{1}{\log n}\sum _{k=1}^{n}\frac{\alpha_{k}}{k}\leq1+2\frac{1-\varPhi(x)}{\varPhi (x)-\varPhi(0)} \quad \mbox{a.s.} $$

On the other hand, if $\widetilde{p}_{k}\neq0$, then we have

4.20 $$\begin{aligned} & \frac{1}{p_{k}}\mathrm{I} \biggl\{ a_{k}\leq \biggl( \frac{\prod_{j=1}^{k}S_{j}}{k!\mu^{k}} \biggr)^{1/(\gamma\sigma_{1} \sqrt{k})}< b_{k} \biggr\} \\ &\quad \geq \frac{1}{\widetilde{p}_{k}}\mathrm{I}\{\widetilde{a}_{k}\leq U_{k}< \widetilde{b}_{k}\} \biggl(1-\frac{p_{k}-\widetilde{p}_{k}}{p_{k}} \biggr) \\ &\quad \geq \widetilde{\alpha}_{k} \biggl(1-\frac{ \mathrm {P}(U_{k}< -x)+\mathrm{P}(U_{k}>x)}{\min \{\mathrm{P}(0\leq U_{k}< x),\mathrm {P}(-x\leq U_{k}< 0) \}} \biggr), \end{aligned}$$

and by Lemma 3.6 and (4.13),

$$ \lim_{k\rightarrow\infty}\frac{ \mathrm{P}(U_{k}< - x)+\mathrm {P}(U_{k}> x)}{\min \{\mathrm{P}(0\leq U_{k}< x ),\mathrm{P}(-x\leq U_{k}< 0) \}}=1-2\frac{1-\varPhi(x)}{\varPhi(x)-\varPhi(0)}. $$

Applying Lemma 3.6, (4.19), and (4.20) implies that

$$ \liminf_{n\rightarrow\infty}\frac{1}{\log n}\sum _{k=1}^{n}\frac{ \alpha_{k} }{k}\geq1-2\frac{1-\varPhi(x)}{\varPhi(x)-\varPhi(0)} \quad \mbox{a.s.} $$

Hence

4.21 $$\begin{aligned} 1+2\frac{1-\varPhi(x)}{\varPhi(x)-\varPhi(0)} \geq& \limsup_{n\rightarrow\infty}\frac{1}{\log n}\sum _{k=1}^{n}\frac{ \alpha_{k} }{k} \\ \geq&\liminf_{n\rightarrow\infty}\frac{1}{\log n}\sum _{k=1}^{n}\frac{ \alpha_{k} }{k} \\ \geq&1-2\frac{1-\varPhi(x)}{\varPhi(x)-\varPhi(0)} \quad \mbox{a.s.} \end{aligned}$$

By the arbitrariness of *x*, let $x\rightarrow\infty$ in (4.21), we have

$$ 1\geq\limsup_{n\rightarrow\infty}\frac{1}{\log n}\sum _{k=1}^{n}\frac{ \alpha_{k} }{k}\geq\liminf _{n\rightarrow\infty}\frac{1}{\log n}\sum_{k=1}^{n} \frac{ \alpha_{k} }{k}\geq1 \quad \mbox{a.s.} $$

Thus

$$ \lim_{n\rightarrow\infty}\frac{1}{\log n}\sum_{k=1}^{n} \frac{ \alpha_{k} }{k}=1 \quad \mbox{a.s.} $$

This completes the proof of Theorem 2.1. □

### Proof of Lemma 3.2

Let $\sigma_{k,l}^{2}:=\operatorname{Var}(\sum_{j=k+1}^{l}b_{j,l}Y_{j})$. First, we prove that

4.22 $$ \sigma_{k,l}^{2}=2(l-k)\sigma_{1}^{2} \bigl(1+o(1)\bigr), $$

where *k* and *l* satisfy (3.3). Note that $\{Y_{n},n\geq1\}$ is a strictly stationary NA sequence with $\mathrm{E}(Y_{1})=0$ and $\operatorname{Var}(Y_{1})=1$, we have

4.23 $$\begin{aligned} \sigma_{k,l}^{2} =&\sum _{i=k+1}^{l}b_{i,l}^{2}+2\sum _{i=k+1}^{l-1}\sum _{j=i+1}^{l}b_{i,l}b_{j,l} \operatorname {Cov}(Y_{i},Y_{j}) \\ =&\sum_{i=k+1}^{l}b_{i,l}^{2}+2 \sum_{i=k+1}^{l-1}\sum _{j=1}^{l-i}b_{i,l}b_{i+j,l} \operatorname{Cov}(Y_{1},Y_{j+1}) \\ =&\sum_{i=k+1}^{l}b_{i,l}^{2}+2 \sum_{j=2}^{l}\sum _{i=1}^{l-k-j+1}b_{k+i,l}b_{k+i+j-1,l} \operatorname{Cov}(Y_{1},Y_{j}) \\ =&\sum_{i=k+1}^{l}b_{i,l}^{2}+2 \sum_{j=2}^{l} \Biggl(\sum _{i=1}^{l}-\sum_{i=l-k-j+2}^{l} \Biggr) \bigl(b_{k+i,l}^{2}-b_{k+i,l}b_{k+i,k+i+j-2} \bigr)\operatorname{Cov}(Y_{1},Y_{j}) \\ =&\sum_{i=k+1}^{l}b_{i,l}^{2}+2 \sum_{j=2}^{l}\sum _{i=1}^{l}b_{k+i,l}^{2} \operatorname{Cov}(Y_{1},Y_{j})\\ & {}-2\sum _{j=2}^{l}\sum_{i=l-k-j+2}^{l}b_{k+i,l}^{2} \operatorname{Cov}(Y_{1},Y_{j}) \\ & {}-2\sum_{j=2}^{l}\sum _{i=1}^{l-k-j+1}b_{k+i,l}b_{k+i,k+i+j-2} \operatorname{Cov}(Y_{1},Y_{j}). \end{aligned}$$

By elementary calculations, under condition (3.3), we obtain

4.24 $$\begin{aligned} \sum_{i=k+1}^{l}b_{i,l}^{2} =& \sum_{i=k+1}^{l} \Biggl(\sum _{j=i}^{l}1/i \Biggr)^{2} \\ =&\bigl(2l-2k-k \log^{2} l\bigr) \bigl(1+o(1)\bigr) \\ =&2(l-k) \bigl(1+o(1)\bigr). \end{aligned}$$

Thus, by (4.23) and (4.24), we get

4.25 $$\begin{aligned} \biggl\vert \frac{\sigma_{k,l}^{2}}{2(l-k)}-\sigma_{1}^{2} \biggr\vert \leq &\frac{1}{l-k}\sum_{j=2}^{l} \sum_{i=l-k-j+2}^{l}b_{k+i,l}^{2} \bigl\vert \operatorname{Cov}(Y_{1},Y_{j}) \bigr\vert \\ &{}+\frac{1}{l-k}\sum_{j=2}^{l} \sum_{i=1}^{l-k-j+1}b_{k+i,l}b_{k+i,k+i+j-2} \bigl\vert \operatorname {Cov}(Y_{1},Y_{j}) \bigr\vert \\ &{}+2\sum_{j=l+1}^{\infty} \bigl\vert \operatorname{Cov}(Y_{1},Y_{j}) \bigr\vert \\ :=&I_{1}+I_{2}+I_{3}. \end{aligned}$$

By the condition of (1.1), for some $\varepsilon>0$, we have

4.26 $$\begin{aligned} I_{1} \leq&c\frac{\log^{2}l}{l-k}\sum _{j=2}^{l}(k+j-1)\frac {1}{(j-1)\log^{2+\varepsilon}(j-1)}\leq c \frac{\log^{2}l}{l-k}\frac {l}{\log^{2+\varepsilon}(l)} \\ \leq&c(\log l)^{-\varepsilon}\rightarrow0, \quad \mbox{as } n\rightarrow\infty, \end{aligned}$$

4.27 $$\begin{aligned} I_{3} \leq&c(\log l)^{-1-\varepsilon}\rightarrow0, \quad \mbox{as } n \rightarrow\infty. \end{aligned}$$

And

4.28 $$\begin{aligned} I_{2} =&c\frac{1}{l-k}\sum _{j=2}^{l} \Biggl[\sum_{i=1}^{l-k-j+1}b_{k+i,k+i+j-2} \sum_{p=k+i}^{l}\frac{1}{p} \Biggr] \bigl\vert \operatorname{Cov}(Y_{1},Y_{j}) \bigr\vert \\ =&c\frac{1}{l-k}\sum_{j=2}^{l} \Biggl[\sum_{p=k+1}^{l}\frac {1}{p} \sum_{i=1}^{(p-k)\bigwedge(l-k-j+1)}b_{k+i,k+i+j-2} \Biggr] \bigl\vert \operatorname{Cov}(Y_{1},Y_{j}) \bigr\vert \\ =&c\frac{1}{l-k}\sum_{j=2}^{l} \Biggl[\sum_{p=k+1}^{l-k-j}\frac{1}{p} \sum_{i=1}^{p-k}b_{k+i,k+i+j-2}+ \sum _{p=l-k-j+1}^{l}\frac{1}{p}\sum _{i=1}^{l-k-j+1}b_{k+i,k+i+j-2} \Biggr] \bigl\vert \operatorname{Cov}(Y_{1},Y_{j}) \bigr\vert \\ :=&c\frac{1}{l-k}\sum_{j=2}^{l} [I_{21}+I_{22} ] \bigl\vert \operatorname{Cov}(Y_{1},Y_{j}) \bigr\vert , \end{aligned}$$

where

$$\begin{aligned} I_{21} =&\sum_{p=k+1}^{l-k-j} \frac{1}{p}\sum_{i=1}^{p-k} \sum _{q=k+i}^{k+i+j-2}\frac{1}{q} \\ \leq& \sum _{p=k+1}^{l-k-j}\frac{1}{p}\sum _{q=k+1}^{p+j-2}\frac{j-1}{q} \\ \leq&\sum _{p=k+1}^{l-k-j}\frac{j-1}{p}\log(p+j-2), \end{aligned}$$

and

$$\begin{aligned} I_{22} =&\sum_{p=l-k-j+1}^{l} \frac{1}{p}\sum_{i=1}^{l-k-j+1} \sum _{q=k+i}^{k+i+j-2}\frac{1}{q}\\ \leq& \sum _{p=l-k-j+1}^{l}\frac {1}{p}\sum _{q=k+1}^{l-1}\frac{j-1}{q} \\ \leq&\sum _{p=l-k-j+1}^{l}\frac {j-1}{p}\log l. \end{aligned}$$

Hence, by (4.23), we get

4.29 $$\begin{aligned} I_{2} \leq&c\frac{1}{l-k}\sum _{j=2}^{l} \sum_{p=l-k-j+1}^{l} \frac {j-1}{p}(\log l) \bigl\vert \operatorname{Cov}(Y_{1},Y_{j}) \bigr\vert \\ \leq& c\frac{1}{l-k}\sum_{j=2}^{l} (j-1)\frac{\log^{2}l}{(j-1)\log^{2+\varepsilon}(j-1)} \\ \leq&c \frac{\log^{2}l}{l-k} \sum_{j=2}^{l} \frac{1}{\log^{2+\varepsilon }(j-1)}\leq c \frac{\log^{2}l}{\log^{2+\varepsilon}l}\rightarrow0, \quad \mbox{as } n \rightarrow\infty. \end{aligned}$$

Equation (4.22) immediately follows from (4.25), (4.26), (4.27), and (4.29).

Let $a_{l,j}=b_{j,l}/\sigma_{k,l}, k+1\leq j\leq l, l\geq1$. Obviously, $\operatorname{Var}(\sum_{j=1}^{l}a_{l,j}Y_{j})=1$ and $\sum_{j=l+1}^{\infty}|\operatorname{Cov}(Y_{1}, Y_{j})|\rightarrow0$ as $l\rightarrow\infty$ by (1.1). Note that $\sigma _{k,l}^{2}=2(l-k)\sigma_{1}^{2}(1+o(1))$, hence by (4.24) we have $\sup_{l}\sum_{j=k+1}^{l}a_{nj}^{2}<\infty$ and $\max_{k+1\leq j\leq l}|a_{lj}|\rightarrow0$ as $l\rightarrow\infty$. Hence (3.4) is satisfied by applying Lemma 3.1.

This completes the proof of Lemma 3.2. □

### Proof of Lemma 3.4

By the condition of (2.9), we have

4.30 $$\begin{aligned} \sum_{\mathcal{H}}\frac{1}{kl(l-k)^{1/5}p_{l}} \leq&c\sum _{l=1}^{n}\frac{(\log l)^{\delta_{1}}}{l (l-l/(\log l)^{2+\delta_{3}} )^{1/5}}\sum _{k=1}^{l}\frac{1}{k} \\ \leq&c \sum_{l=1}^{n} \frac{(\log l)^{1+\delta_{1}}}{l^{1/5}}=O\bigl((\log n)^{2-\epsilon}\bigr). \end{aligned}$$

It proves (3.7). The proofs of (3.8) and (3.9) are similar to the proof of (3.7). By using Markov’s inequality, (4.22), and $\varepsilon _{l}=1/(\log l)^{\delta_{4}}$, we have

4.31 $$\begin{aligned} &\mathrm{P} \biggl\{ \biggl\vert \frac{1}{\sigma_{1}\sqrt{2l}}S_{k,k} \biggr\vert \geq \varepsilon _{l} \biggr\} \leq\frac{\operatorname{Var}(S_{k,k})}{2l\sigma _{1}^{2}\varepsilon_{l}^{2}}\leq \frac{2k\sigma_{1}^{2}}{2l\sigma_{1}^{2}\varepsilon _{l}^{2}}=\frac{k}{l}(\log l)^{2\delta_{4}}, \end{aligned}$$

4.32 $$\begin{aligned} &\mathrm{P} \biggl\{ \biggl\vert \frac{1}{\sigma_{1}\sqrt{2l}}b_{k+1,l}\widetilde {S}_{k} \biggr\vert \geq\varepsilon _{l} \biggr\} \leq \frac{b_{k+1,l}^{2}\operatorname{Var}(\tilde {S}_{k})}{2l\sigma_{1}^{2}\varepsilon_{l}^{2}}\leq\frac{(\sum_{i=k+1}^{l})^{2}k}{2l\sigma_{1}^{2}\varepsilon_{l}^{2}}\leq c \frac{k}{l}(\log l)^{2+2\delta_{4}}. \end{aligned}$$

Noting the condition of $0<\epsilon<1-2(\delta_{1}+\delta_{4})$, we get

4.33 $$\begin{aligned} \sum_{\mathcal{H}}\frac{1}{klp_{k}p_{l}} \frac{k}{l}(\log l)^{2+2\delta_{4}} \leq&\sum_{l=1}^{n} \frac{(\log l)^{2+\delta_{1}+2\delta _{4}}}{l^{2}}\sum_{k=1}^{l/(\log l)^{2+\delta_{3}}}(\log k)^{\delta_{1}} \\ < &\sum_{l=1}^{n}\frac{(\log l)^{2+2\delta_{1}+2\delta_{4}}}{l^{2}} \frac{l}{(\log l)^{2+\delta_{3}}} \\ \leq&\sum_{l=1}^{n}\frac{(\log l)^{2\delta_{1}+2\delta_{4}-\delta _{3}}}{l}=O \bigl((\log n)^{2-\epsilon}\bigr). \end{aligned}$$

It proves (3.10) and (3.11). By (4.12), we have

4.34 $$ \mathrm{P}\bigl\{ \vert T_{l} \vert \geq\varepsilon_{l} \bigr\} \leq\frac{2\sqrt{2}\gamma\sum_{i=1}^{l}\frac{1}{i}}{\sigma_{1}\sqrt{l}\varepsilon_{l}}\leq c \frac{(\log l)^{1+\delta_{4}}}{l^{1/2}}. $$

Thus

4.35 $$\begin{aligned} \sum_{\mathcal{H}}\frac{1}{klp_{k}p_{l}}\mathrm{P} \bigl\{ \vert T_{l} \vert \geq\varepsilon _{l} \bigr\} \leq&c\sum_{l=1}^{n}\frac{(\log l)^{1+\delta_{1}+\delta _{4}}}{l^{3/2}}\sum _{k=1}^{l}\frac{(\log k)^{\delta_{1}}}{k}\leq c\sum _{l=1}^{n}\frac{(\log l)^{2+2\delta_{1}+\delta_{4}}}{l^{3/2}} \\ \leq&c \sum_{l=1}^{n} \frac{(\log l)^{1+2\delta_{1}+\delta_{4}}}{l}=O\bigl((\log n)^{2-\epsilon}\bigr). \end{aligned}$$

It proves (3.12). This completes the proof of Lemma 3.4. □

## Conclusions

In this paper, we study the almost sure local central limit theorem (ASLCLT) for products of partial sums of negatively associated random variables. The obtained results extend the theorem of Weng et al. [21] for i.i.d. random variables to NA random variables, and it is a generalization of the result given by Jiang and Wu [24] from partial sums to products of partial sums under NA random variables. The main idea of the proofs relies on estimate of the covariance structure of the underlying NA sequence. It is a classic and effective technique for this kind of the problem.

Matuła and Stȩpień [16] provided a very mild assumption on the summability on covariances to obtain limit theorems (CLT and ASCLT). As we all know, the ASLCLT is a general result which contains the ASCLT. In this paper, the optimality of the assumptions of Theorem 2.1 is not discussed, in particular assumptions (1.1), (1.2), and (2.8). This will be another interesting topic of research, and we will leave this topic for the future.
